# Analysis of Why Alzheimer’s Dementia Never Spontaneously Reverses, Suggests the Basis for Curative Treatment

**DOI:** 10.3390/jcm12144873

**Published:** 2023-07-24

**Authors:** Jeffrey Fessel

**Affiliations:** Department of Medicine, University of California, 2069 Filbert Street, San Francisco, CA 94123, USA; jeffreyfessel@gmail.com; Tel.: +1-415-563-0818

**Keywords:** Alzheimer’s dementia, MCI, spontaneous reversal absent/present, cure, treatment, TGF-β, Wnt/catenin-β, EMT, concurrence with comorbid risk factors

## Abstract

A paradox regarding Alzheimer’s dementia (AD) and mild cognitive impairment (MCI) is thats spontaneous cure of AD has never been reported, whereas spontaneous cure for MCI occurs fequently. This article analyzes what accounts for this difference. It holds that it is not merely because, for any condition, a stage is reached beyond which it cannot be reversed, since even widely metastatic cancer would be curable were there effective chemotherapy and rheumatoid arthritis became controllable when immune-suppressant treatment was introduced; thus, so could AD be reversible via effective therapy. The analysis presented leads to an explanation of the paradox that is in four categories: (1) levels of transforming growth factor-β are significantly reduced after the transition from MCI to AD; (2) levels of Wnt/β-catenin are significantly reduced after the transition; (3) there is altered epidermal-mesenchymal transition (EMT) in neurons after the transition; (4) there may be risk factors that are either newly operative or pre-existing but worsened at the time of transition, that are particular to individual patients. It is suggested that addressing and ameliorating all of those four categories might cure AD. Medications to address and ameliorate each of the four categories are described.

## 1. Introduction

A major mystery affects Alzheimer’s dementia (AD). Despite its prevalence of 50 million cases worldwide, about 10% of whom are in the U.S., there is not one report of spontaneous reversion to normal cognition. That is a mystery because such reversion is frequent in cases of mild cognitive impairment (MCI). For 1873 individuals with MCI in three cohort studies, Li et al. showed a 50.8% rate of reversion to normal cognition after an average follow-up of 4.4 years [1]; for a different cohort of 1208 subjects with MCI, Pandya et al. showed 35% reversion to normal cognition at three years [2]. Overton et al. analyzed data for 331 participants, aged 60–95 years with MCI, and found that after 6 years, 58% had reverted to normal cognition; and among those patients who had a follow-up of 12 years, 76% continued to have normal cognition [3]. Reports of reversions of MCI to normal cognition, whose timing was not provided, gave similar rates [4,5]. Parenthetically and unsurprisingly, individuals who had reverted to normal cognition were only about half as likely to be carriers of the *APOE4ε* gene allele than were those who progressed to AD. The studies showed that those who reverted were better educated; molecular biology explaining the benefit of education is unclear.

If the factors responsible for preventing reversion to normal cognition in patients with AD were explained, then treatment directed at those factors might be curative. Although it may be that for any condition a stage is reached beyond which it cannot be reversed, that explanation does not hold for AD because of examples where apparently incurable conditions become curable with the advent of better treatment, e.g., rheumatoid arthritis that became curable after immune-suppressant therapy was introduced; and even worst-case, widely metastatic cancer, would be curable if there were effective chemotherapy. The same holds for AD, i.e., it is not beyond the reach of curative therapy. The purpose of this article is to suggest a cure for AD based upon the tentative explanation of the above paradox, which, expectedly, is multifactorial and complex: the barrier to curing AD is the combined effects of decreased levels of transforming growth factor beta (TGF-β) and of Wnt/β-catenin signaling, an alteration of epithelial-mesenchymal transition (EMT), plus important risk factors particular to each patient in the year preceding the transition from MCI to AD. Addressing all of those factors could produce a cure for AD.

## 2. Level of TGF-β and Its Receptors in AD: The Role of Smads; the Importance of TGF-β Isotypes; and Their Differential Presence in Neurons and Astrocytes

While the absolute level of TGF-β is not reduced in MCI [6,7], in AD it was approximately 30–50% lower than in controls [7,8,9], which may the reason why AD both does not undergo spontaneous remission and is resistant to therapy. However, conflicting data concerning TGF-β have been reported: for example, elevated levels of TGF-β have been seen in both serum and spinal fluid [10,11] Further, some reports show data that are both conflictual and perplexing: Motta et al found that compared with controls, there was an 8-fold increase of plasma TGF-β in mild AD, only a 3.5-fold increase in moderate AD, but a 75% decrease in severe AD [12]; and in the entorhinal gyrus, superior temporal gyrus, and occipital cortex, Luterman et al saw approximately equal levels of TGF-β in those with CDR score between 0.5 and 4 but increased levels in those with CDR scores of 5 [13].

The conflictual data have several possible explanations, an important one being that activated TGF-β binds to specific heteromeric transmembrane receptor complexes that transduce the signal intracellularly via effector proteins termed Smads, and the Smad pathway is the major downstream consequence of activated TGF-β. When activated by TGF-β, the Smad complex moves to the nucleus where it regulates target gene transcription [14]. There, specific Smads become phosphorylated and associate with other Smads, and these Smad complexes accumulate in the nucleus, where they modulate the expression of target genes [15]. Among the Smads, Smad2 has a role in maintaining neurons in an activated state [16]. However, Lee et al and Ueberham et al, both showed that in AD there is reduction of Smad2 in neuronal nuclei [14,16]. Ueberham et al used labeled antibodies that detected phosphorylated serines of five different groupings within Smad2 and compared their results in five AD patients all with Braak stage 5–6, and five controls all with Braak stage 0 [16]. Phosphorylations of Smad2 at serine 465/467 gave a nuclear signal in 96.4% of controls but only in 70.1% of the AD patients (*p* < 0.001); cytoplasmic granules with a nuclear signal was seen in no controls but in 11.7% of AD patients (*p* < 0.001); and cytoplasmic granules without a nuclear signal were seen in 0.1% of controls but in 10.6% of AD patients (*p* < 0.001). Similar data were reported by Lee et al. [14]. The relevance of these data regarding the conflictual reports about the levels of TGFβ in AD, is that in AD a substantial amount of the Smad complex does not reach the nucleus despite high serum and spinal fluid levels of TGF-β; therefore those high levels have fewer downstream consequences, i.e., functional effect, than if the entire Smad complex had entered the nucleus. It is likely, also, that the high serum levels of TGF-β are compensatory to the reduced downstream effect, via inhibitory Smads, including Smad6 and Smad7, that are major, negative regulators of TGF-β signaling via forming negative feedback loops [17,18].

Another reason for variable data regarding levels of TGF-β, is the need to differentiate between the three isotypes of TGF-β and its receptors, and whether TGF-β was seen in neurons or glia. Peress studied cases of AD for the three isotypes TGF-β1, 2, & 3 [19]. They saw TGF-β1in neurites within senile plaques, TGF-β2 in neurofibrillary tangles, plaque neurites, microglia, and astrocytes, and TGF-β3 in Hirano bodies (these are cytoplasmic inclusions composed mainly of actin and actin-associated proteins). Ren et al. treated human neuronal cells with antibodies to TGF-β1 or 2, which produced both increased cell viablility and reduced apotosis [20]. Mocali et al found that plasma from AD subjects had reduced levels of TGF-β1 [9]; likewise, Huang et al saw a 30% reduction of TGF-β1 serum levels in AD patients but a non-significant reduction in serum from MCI patients [7] and those levels were found by Motta et al to vary by AD stage—viz, 8-fold higher in mild, 3.8-fold higher in moderate, and slightly reduced in severe stage disease [12]. Lippa et al saw little difference between AD patients and controls for immune staining of neurons for TGF-β receptors 1&2 but glia from patients with AD showed increased reactivity [21]. Finally, Zetterberg et al saw significantly higher spinal fluid levels of TGF-β1 in AD than in controls [11].

A consideration, never accounted for in reports of results that are, only, apparently conflictual (because the fall of an arrow is as musc a segment if its arc as is the rise), are the effects of the concurrent use of medications. Some drugs raise levels of TGF-β, e.g., aspirin and statins, used either separately or together ([22,23,24]); other drugs may lower levels of TGF-β, e.g., losartan [25], and chloroquine, hydroxychloroquine, amodiaquine, and azithromycin which use a mechanism that reduces production of mature bioactive TGF-β [26].

In brief: TGF-β and its isotypes have levels in AD that are variable but often explicable; overall, they are mostly reduced. Raising those levels in conjunction with other measures described in this article, might contribute to curing AD.

## 3. Wnt/β-Catenin Signaling and Cognition Are Negatively Correlated and Contribute to Explaining the Reversibility of MCI and the Irreversibility of AD

In the brain, Wnt/β-catenin signaling promotes neuronal survival and neurogenesis, and its loss makes neurons more susceptible to apoptosis. Its activation rescued both the neuronal apoptosis and the blood–brain barrier (BBB) dysfunction caused by Aβ [27]. Studies by Inestrosa et al. showed that Wnt/β-catenin is involved in regulating synaptic plasticity and maintaining BBB integrity; Inestrosa et al. also found that activated WNT/β-catenin signaling prevented neural toxicity caused by Aβ; that WNT/β-catenin participates in a normal degree of tau phosphorylation and in learning and memory; and that WNT/β-catenin dysfunction results in Aβ production and aggregation [28]. Tay et al. followed 14 subjects with MCI and 74 with mild to moderate AD and measured the scores for the Clinical-Dementia-sum of boxes (CDR-SB) at baseline and after one year and assessed the correlations between changes in the CDR-SB and serum levels of Dickkopf-1 (Dkk-1), which is an antagonist of Wnt [29]. Decreased levels of Wnt, as shown by the increase in Dkk-1, were significantly associated with progressively higher CDR-SB scores (indicating more impairment) among patients with AD but not among patients with MCI. Confirmatory data relating to levels of Wnt7B in brain samples were seen by Folke et al. [30]. Dkk-1 serum levels at baseline were not significantly different between progressors and non-progressors but at one year, those who progressed had Dkk-1 serum levels of 1021 pg/mL versus 731 pg/mL in the non-progressors (*p* = 0.024). They also saw a linear correlation between the decrement in Wnt7B levels and Braak stages 1, 2, 3, 5, 6 (r = 0.794; *p* < 0.001); the controls had Braak stages 1–3, the AD patients had Braak stages 5 and 6. Decrements of Wnt7B levels correlated with decreasing MMSE scores (r = 0.795; *p* < 0.003). 

WNT/β-catenin is also involved in the maintenance of the BBB (reviewed by Erickson and Banks [31]; although the data are conflictual, the bulk of the evidence indicates that in AD, there is disruption of the BBB that participates in the complex pathogenesis of AD [31]. As shown by Montagne et al., this is an early event because there is an age-dependent BBB breakdown in the hippocampus, which in its CA1 and dentate gyrus subdivisions was worsened in MCI [32]. As reported by Halleskog et al., Wnt/β-catenin is involved in several ways: the AD brain contains inflammatory cytokines such as IL-1, IL-6, and TNF-α, largely derived from activated microglia that contain increased β-catenin [33]; in the presence of Wnt, there is stabilization of β-catenin that become transported to the nucleus where it induces expression of target genes such as *claudin-3*, *GLUT-1*, *PDGF-B*, and *P-gp*, that maintain the integrity of the BBB. Others confirmed that Wnt ligands participate in the repair of the BBB [34]. However, the decreased Wnt levels in AD [29] must disfavor the BBB [35]. Because microglia are part of the neurovascular unit, they are in close contact with the BBB. 

In brief, reports demonstrate that Wnt levels are approximately normal in patients with MCI but are substantially decreased in patients with AD, which strongly suggests, in view of the importance of Wnt/β-catenin signaling for brain function, that its decreased levels are related to the causation of the transition from MCI to AD and to the anomalous absence of spontaneous reversion to normal cognition in AD.

## 4. The Effects of EMT

EMT refers to a differentiation process by which cells change their phenotype from being one that primarily typifies cells of epithelial lineage (E) to one that typifies cells of mesenchymal (M) lineage. The direction may be either E-to-M (EMT) or M to E (MET). Many studies show the effects of EMT on the development of the embryonic brain [36], for the morphogenesis of which the cadherin/catenin adhesion system plays a key role. Cadherins are involved in the formation and maintenance of the neuroepithelium, neurite extension, and migration of neuronal cells [37]. Although there are many cadherins, the majority of neurons in the brain either represent the E phenotype and express E-cadherin or represent the M phenotype and express N-cadherin. 

In AD, there is a down-regulation of neurons with the M phenotype. This was shown by Liu et al., who immuno-stained FAM3C, a key molecule in causing the E-to-M transition, and found it 45% lower in AD brains than in controls [38]. Those findings were confirmed in studies by Watanabe et al., who also saw an overall 46% reduction of FAM3C; it was 27% reduced in Braak stages 3–4 as compared with non-demented controls having Braak stages 1–2 but was 51% reduced in Braak stages 5–6 [39]. Hasegawa et al. demonstrated that TGF-β induced the neuronal expression of FAM3C [40]. Thus, the treatments described below that increase the levels of TGF-β may lead to an increase in FAM3C, then of N-neurons, and finally to neurogenesis that should benefit the attempt to reverse AD. In addition, Hasegawa et al. also found that FAM3C substantially decreased the secretion of both Aβ_40_ and Aβ_42_ (*p* < 0.001). 

Although, at this time, there are no data showing that increasing M-neurons gives functional benefit, the issue is relevant to the present article because TGF-β drives E-to-M, and the levels of TGF-β become reduced after the transition from MCI to AD; it seems possible that the consequent reduction of E-to M relates to why MCI is often spontaneously reversed but that never happens once AD occurs. 

In the nervous system, cadherin-based sorting defines axon tracts and may encode certain aspects of synapse specificity; afferents and their targets have the expression of a particular cadherin in common, providing a recognition code of homophilic cadherin binding [41]. E-cadherins are expressed in the adult forebrain germinal zones and promote neurogenesis; adult neural stem cell colonies (NSC) were decreased dose-dependently via E-cadherin antibodies [42]. Involvement of genetic interactions in this process is illustrated by the fact that presenilin 1 (PS1), mutants of which produce a familial AD, binds directly to E-cadherin; PS1 displaces p120 from E-cadherin, stabilizes the binding of β- and γ-catenins to E-cadherin, increases linkage of the cadherin/catenin complex to the cytoskeleton, and stimulates Ca^2+^- and cadherin-dependent cell–cell aggregation [43].

Using an epithelial cell line, Piek et al. showed that TGF-β evoked mesothelial transition and that this was via Smad receptors [44]. N-cadherin maintains the architecture of neural tissues and, like E-cadherin, regulates the proliferation and differentiation of NSC. In the hippocampus, N-cadherin regulated mossy fiber fasciculation and was essential for CA3 dendrite arborization [41]. In cultures of cerebellar neurons, there was a linear relationship between N-cadherin levels and the length of neurites [45]. Kon et al. showed that N-cadherin binds fibroblast growth factor receptors (FGFR); as a result, FGFRs accumulate and stimulate prolonged phosphorylation and activation of Erk1/2 [46]. ERK1⁄2 can promote either neuronal survival or neuronal death, depending upon both the magnitude and the duration of ERK1⁄2 activation [47]. The levels of ERK1/2 in cerebral spinal fluid (CSF) were 77% higher in patients with AD than in controls, whereas they were only 22% higher in those with MCI [48]. Since, as compared with controls, the levels of ERK1/2 were greatly increased in the CSF of patients with AD but far less so in the CSF of patients with MCI, and TGF-β is normal in MCI but greatly reduced in AD, one may infer that the rise in ERK1/2 resulted from the low level of TGF-β and should not worsen by raising that level.

No data exist which allow estimation of either the direction of the change or the resulting percentage amounts that affect each lineage as a result of the decrement in TGF-β level that occurs when MCI transitions to AD. Nevertheless, it is quite possible that the changes in percentages of E-cadherin-expressing neurons versus N-cadherin-expressing neurons in the brain are involved in the apparent resistance of patients with AD to undergo reversal to normal cognition. It is reasonable to presume that the cause of those changes is the reduced level of TGF-β; therefore, raising the level of TGF-β will benefit whatever change affects EMT in patients with AD.

## 5. The Effects of Factors Peculiar to Individual Patients That Contribute to Determining the Transition from MCI to Dementia

That AD is caused by specific events that may be particular to every patient is one of several reasons why uniform treatment has never succeeded. An example of patient-specific events that produced irreversibility after conversion from mild cognitive impairment (MCI) to AD was seen in a brother-sister pair within a large Columbian kindred that carries the *PSEN1* gene with the E280A mutation, which overproduces amyloid-β_42_ (Aβ_42_) [49,50]. Members of that kindred develop dementia at the median age of 49 years, but that was delayed by almost 25 years in the two siblings, each of whom had a heterozygous variation in the *RELN* gene that was absent when sought in other, affected members of the kindred. The sister, in addition to having the variation in the *RELN* gene, also had two copies of the rare Christchurch (*APOEch*) R136S mutation at codon 154 of *APOE3*. A single copy of this *APOE3ch* mutation was seen in only 7 (6%) of 117 kindred members. This R136S mutation decreases the ability to trigger Aβ_42_ aggregation and may have been another factor delaying the sister’s AD [50]. 

Thus, a fourth modifiable element that might contribute to initiating the progression of MCI to AD is a risk factor that is either newly present or pre-existing but worsened at the time of transition to AD. What this element might comprise has been examined in three analyses, the most recent by the Lancet Commission [51] and by two earlier ones [52,53]. Twelve risk factors for AD that affect ~40% of the worldwide AD population were identified by the Lancet Commission; they included inadequate exercise, hearing loss, infrequent social contact, plus six others for which Barnes and Yaffe gave the following population prevalences: physical inactivity 32.5%, smoking 20.6%, depression 19.2%, mid-life hypertension 14.3%, mid-life obesity 13.1%, and diabetes 8.7% [52]. Pal et al. analyzed 12 reports involving 6865 participants and emphasized that metabolic syndrome comorbid with MCI is also a risk factor for progression to AD [53]; that was confirmed by Solfrizzi et al. in the Italian Longitudinal Study on Aging, in which MCI patients with metabolic syndrome had an approximately doubled risk of progression to dementia as compared with those subjects without that syndrome [54].

In brief, if at the time when an individual with MCI becomes identified as having progressed to dementia, there is also the occurrence during either the past or future year of one or more of the eleven conditions indicated in this paragraph, correction of that comorbidity plus enhancing the levels of TGF-β and Wnt/β-catenin and correcting the altered EMT might cure dementia.

### 5.1. Overlaps between the Categories of Causes

There are overlaps between three of the four categories of causes: Wnt/β-catenin, TGF-β, and EMT. Both Wnt/β-catenin and TGF-β decrease when MCI transitions to AD, and each has enhanced levels induced using doxycycline [55]. TGF-β has a role in EMT, as shown by its induction of EMT [56] via mechanisms that include enhancement of FAM3C expression [40,57]. Wnt/β-catenin also enhances EMT: addition of Wnt-1 to normal epithelial cell lines stabilizes the complex between cytoplasmic β-catenin and lymphoid enhancing factor-1 (LEF-1) that then transports to the nucleus, leading to EMT, and Kim et al. demonstrated that overexpressed LEF-1 dramatically promoted EMT [58].

### 5.2. Genetic Factors

The high rate of dementia in patients with type 2 diabetes (T2DM), which has polygenetic risk factors, is interesting because it is bidirectional, i.e., T-DM patients have more AD, but AD subjects have more T2DM. In a systematic review of 13 longitudinal, population-based studies, Biessel et al. found an increased risk of AD in patients with DM [59] Janson et al. saw that in AD versus non-AD control subjects, there was an increase in both T2DM (35% vs. 18%; *p* < 0.05) and preDM (46% vs. 24%; *p* < 0.01), so 81% of cases of AD had either T2DM or preDM [60]. In AD, this is a very early event because the slope of increased fasting blood glucose after age 10 years was also greater in AD than in non-AD controls (*p* < 0.01), and islet amyloid was more frequent (*p* < 0.05), and extensive (*p* < 0.05) in patients with AD than in control subjects. Unsurprisingly, possession of the APOEɛ4 allele increased the risk of AD by 35% in those with DM [61].

## 6. Treatments to Ameliorate the Four Categories of Causes That Determine the Transition from MCI to AD, and If Combined, Might Cure Dementia

The conclusion from the above account is that four reasons may explain the apparent mystery that the likelihood of spontaneous reversion to normal cognition becomes virtually zero once the process of impaired cognition has moved beyond MCI to AD, whereas spontaneous reversion is frequent in MCI. Those reasons are:

(1) levels of transforming growth factor-β are significantly reduced after the transition from MCI to AD;
(2) levels of Wnt/β-catenin are also significantly reduced after the transition;
(3) there is altered epidermal-mesenchymal transition (EMT) in neurons after the transition;
(4) newly operative risk factors may be present at the time of transition that are particular to individual patients.

## 7. Treatments That Raise Levels of TGF-β

TGF-β promotes neurogenesis, and neural stem cells are present in the subventricular zone (SVZ), into which Mathieu et al. injected adenoviral vectors expressing TGF-β; three weeks later, they saw increased neurogenesis in the ipsilateral hemisphere [62]. In organs such as kidney and breast, activation of the TGF-β signaling pathway inhibits cell proliferation and increases extracellular matrix production, so Miettinen et al. used mammary epithelial cells to demonstrate that TGF-β can induce epithelial-to-mesenchymal transition [63]. As regards the inadequate TGF-β levels in AD, there are several available drugs to raise them. Knowing that TGF-β levels increase after selective serotonin reuptake inhibitors (SSRI) such as fluoxetine are administered to depressed patients, Caraci et al. added fluoxetine in therapeutic levels (100 nM–1 µM) to mixed glia-neuronal cultures and saw significant prevention of Aβ-induced neural toxicity; but fluoxetine did not did not prevent such toxicity in pure cultures of neurons [64]. However, it is known that astrocytes produce TGF-β [65]; Caraci et al. then found that fluoxetine-induced astrocytes secrete TGF-β and that the medium in which those astrocytes were cultured protected cultures of neurons from toxicity-induced by Aβ [64]. Confirming the role of astrocytes and the benefit of SSRI treatment, Zepeda et al. found that five days after cerebral infarction, the penumbra of the infarcted area showed that the selective serotonin and norepinephrine reuptake inhibitor (SSNRI) venlafaxine, caused no increase in neuronal levels of TGF-β levels but a 35% increase in astrocytic levels [66]. Memantine is another drug that induces increased production of TGFβ. In a randomized study of patients taking methadone for opiate addiction, Lee et al. gave either low doses of memantine (5 mg daily) or placebo [67]. After 12 weeks, those who had been administered memantine had significantly higher levels of TGF-β and required lower doses of methadone. Finally, after exposure to insulin, TGF-β receptors translocated from intracellular stores to the plasma membrane, thus enhancing responsiveness to TGF-β [68]. Insulin has direct access to the brain if administered intranasally and has been shown as beneficial for AD [69]. In brief, treatments to increase TGF-β could include fluoxetine, venlafaxine, memantine, and intranasal insulin. Finally and as mentioned above, aspirin and statins raise levels of TGF-β ([22,23,24]).

## 8. Treatments That Raise Levels of Wnt/β-Catenin

Three reports show that doxycycline, a commonly used antibiotic, raises levels of Wnt/β-catenin. Noting that Wnt signaling is established as an essential bone-promoting mechanism associated with bone healing, Song et al. showed that both Wnt7b and doxycycline increased the density of callus at the site of a fracture [70]. Zhang et al. also found that doxycycline increased bone formation, and this was accompanied by up-regulation of β-catenin and TGF-β [71]. Gomes et al. reported that doxycycline decreased the immunostaining of Dikkopf-1 (Dkk-1), which is an antagonist of Wnt/β-catenin, by as much as 63% and increased the immunostaining of Wnt-10b by as much as 150% [55].

Niedzielski et al. reviewed reports showing that several statins, including simvastatin, atorvastatin, and rosuvastatin, promote the actions of Wnt/β-catenin, one mechanism for which is the inhibition of Dkk-1 [72]. There are also other mechanisms: Gao et al. found that simvastatin raises levels of the lipoprotein receptor, LRP-6, binding of which by Wnt initiates a number of intracellular signaling cascades; and the anti-apoptotic effect that simvastatin had on cultured neuronal cells was reduced by suppression of the Wnt/β-catenin pathway [73]. Qiao et al. used embryonic stem cells to demonstrate that inhibition of Wnt signaling reduced the release of several transcription factors [74].

Glucocorticoids also enable Wnt because, after disruption of their expression, Zhou et al. noted decreased levels of β-catenin and of Wnt9a and 10b [75].

In brief, several treatments, doxycycline, several statins, and corticosteroids, increase the efficacy of Wnt/β-catenin signaling.

## 9. Treatments to Promote EMT

As indicated above, evidence indicates that treatments raising levels of TGF-β will also raise EMT. 

### 9.1. Treatment for Conditions That Occurred during One Year either before or after the Transition from MCI to Dementia

If, during the year before or after the transition from MCI to dementia, one or more of the comorbid risk factors became either newly present or worse if pre-existing, then addressing/correcting that factor or factors should supplement treatment for alterations in Wnt/β-catenin, TGF-β, and EMT.

### 9.2. Clinical Trial to Validate the Hypothesis That Low Levels of TGF-β and Wnt/Catenin-β Are Responsible for Why Spontaneous Remission Does Not Happen in Established AD

The primary objective of the study is to examine the null hypothesis that low levels of TGF-β and Wnt/catenin-β are not responsible for why spontaneous remission does not happen in established AD. The number of participants needed for the trial would be calculated by a bio-statistician on the basis of the trial medication not producing a cure rate in 10% of those receiving the trial medication and a loss to follow-up of 10% per year. Participants would be randomized to receive either standard of care or a combination of doxycycline, atorvastatin, and pioglitazone with either fluoxetine (if major depression diagnosed at any time) or lithium (if bipolar disease diagnosed at any time). Inclusion criteria include AD diagnosis established within 2 years of baseline; age ≥ 65. Exclusionary criteria include the presence of type 2 diabetes, untreated hepatitis C, trisomy 21, and a history of any major psychiatric disease, allergy, or known intolerance to any of the medications. Current cognition will be measured via abnormal scores in the CDR or MMSE tests. Patients will be seen every 4 weeks for a brief interview, physical examination, and blood tests for routine CBC, liver function tests, and plasma creatinine. Neuropsychological tests will be performed at baseline, every 6 months, and at the end of the study, which will be at 48 weeks after study entry.

## 10. Limitations of the Proposed Approach

One limitation is ascertaining the date when the AD diagnosis was first made for each patient, including whether any of the eleven relevant risk factors for AD was either newly present or, if pre-existing, worsened. The difficulty is that the new or worsened presence of the risk factor might not be evident until after AD becomes established. Therefore, one must look for the occurrence of the risk factor both one year before and one year after the date when the AD diagnosis was first made. That could pose a problem if each of those years were to show different risk factors, each of which requires different treatments. One solution would be to exclude such a patient from participating in a clinical trial for curative therapy because there should be no dearth of other participants. An alternative solution would be to decide, in advance of a clinical trial, to treat the risk factor with the highest risk for AD, as listed in the article by Barnes and Yaffe [52].

Other limitations concern the possible adverse consequences of the suggested treatments. The first of these is that raising levels of TGF-β may lead to raised levels of ERK1/2, and the latter may be either clinically beneficial or clinically deleterious. Pei et al. indicated possibly deleterious consequences by finding phosphorylated (activated) ERK1/2 in the initial stages of neuronal neurofibrillary degeneration of AD brains even before amyloid deposition appeared [76]. On the other hand, the benefit of brain-derived neurotrophic factor (BDNF) on cognitive improvement in rats injected with Aβ_42_ was blocked by an inhibitor of ERK [77]; similarly, such inhibition also blocked the increased density of dendritic spines induced by BDNF [78]. The induction of long-term potentiation via BDNF required the activation of ERK [79], and the beneficial modulation via BDNF of high-frequency synaptic transmission was prevented by MAPK inhibition (note that ERK1/2 are MAPK’s) [80]. Another mechanism that benefits from ERK activation is the degradation of Aβ by astrocytes, which is facilitated by two enzymes in the brain, neprilysin (NEP) and insulin-degrading enzyme (IDE). Yamamoto et al. demonstrated that insulin exposure enhanced the degradation of Aβ via an increased expression by astrocytes of NEP and IDE that was mediated by ERK [81]. Overall, therefore, and in the context of this article, the clinically beneficial consequences of raising levels of ERK1/2 by raising levels of TGF-β are dominant. That is also shown by the observations (1) that cognitive impairment may frequently reverse and become normal in MCI where the level of TGF-β is normal but (2) that does not happen in AD where the level of TGF-β is reduced, and (3) as compared with controls, the levels of ERK1/2 were +77% in the CSF of patients with AD but only +22% in the CSF of patients with MCI [48]. One may infer that any increase in ERK1/2 produced by the low level of TGF-β should not worsen by raising that level and may even reduce. 

Other concerns about adverse reactions from the suggested treatments are that, although TGF-β is often held to be anti-atheroma, some contrary data exist, as reviewed by Toma and McCaffrey [82]; therefore, treatments to raise levels of TGF-β should be avoided in patients with a history of cerebro-vascular disease. Lastly, regarding using statins to raise levels of the Wnt/β-catenin signaling pathway, simvastatin should be avoided because of its multiple side effects. 

## 11. Summary and Conclusions

(1) The goal of treating AD is its cure, i.e., reversal to normal cognition.
(2) A paradox regarding AD and MCI is that spontaneous cure has never been reported for AD, whereas spontaneous cure for MCI occurs frequently. Understanding the reasons for that paradox suggests the formulation of curative therapy.
(3) The explanation of the paradox lies in four categories of factors:
  - levels of TGF-β are significantly reduced after the transition from MCI to AD;
  - levels of Wnt/β-catenin are significantly reduced after the transition from MCI to AD;
  - there is altered epidermal-mesenchymal transition (EMT) in neurons after the transition from MCI to AD;
  - risk factors for AD that are either newly operative or worsened, if pre-existing, may occur close to the time of transition, that are particular to individual patients.
(4) It is suggested that addressing all of those four categories of factors might cure AD. Methods to do so are available for each category and are described. A clinical trial would validate the suggested treatments.

## Data Availability

Not applicable.
